# Coordinate Regulation of DNA Methylation and H3K27me3 in Mouse Embryonic Stem Cells

**DOI:** 10.1371/journal.pone.0053880

**Published:** 2013-01-11

**Authors:** James A. Hagarman, Michael P. Motley, Katla Kristjansdottir, Paul D. Soloway

**Affiliations:** Division of Nutritional Sciences, College of Agriculture and Life Sciences, Cornell University, Ithaca, New York, United States of America; Michigan State University, United States of America

## Abstract

Chromatin is separated into functional domains distinguished by combinatorial patterns of post-translational histone modifications and DNA methylation. Recent studies examining multiple histone modifications have found numerous chromatin states with distinct profiles of chromatin marks and functional enrichments. There are data showing coordinate regulation between DNAme and H3K27me3, which are both involved in the establishment and maintenance of epigenetic gene silencing, but the data are conflicting. Multiple studies have presented evidence to support the theory that PRC2 and DNAme cooperate to achieve silencing, or alternatively that H3K27me3 and DNAme act antagonistically. Here we examine the effect loss of either PRC2 or DNA methyltransferase activity has on the placement of the reciprocal mark in mouse ES cells. We find that DNAme is acting globally to antagonize the placement of H3K27me3, in accordance with recently published results. At least 471,011 domains in the mouse genome acquire H3K27me3 when DNAme is diminished. Of these 466,563 have been shown to be fully methylated in wildtype ES cells, indicating the effects of DNAme on H3K27me3 are direct. In a reciprocal experiment, we examine the effect loss of PRC2 has on the placement of DNAme. In contrast to the global antagonism DNAme has on the placement of H3K27me3, loss of H3K27me3 has a modest effect on DNAme, with only 4% of genes undergoing changes in DNAme, including 861 showing increases and 552 showing losses of overall DNAme. We anticipate that integrating genomic datasets where the effect of loss of a particular epigenetic mark has on the placement of other marks will help elucidate the rules governing epigenetic regulation and what role coordinate regulation of epigenetic marks plays in development and disease.

## Introduction

The genetic information of eukaryotic cells is stored in the nucleus in the form of chromatin. The basic unit of chromatin is the nucleosome, a complex of DNA wrapped around an octamer of core histone proteins. Post-translational modification of DNA and histones, including acetylation, methylation, phosphorylation, ribosylation, glycosylation and ubiquitination separates chromatin into functional domains. Considerable evidence suggests that posttranslational modifications to DNA and histones define a ‘chromatin state’ that dictates a distinct cellular state and thus a particular transcriptional program (Reviewed in [1]–[3]).

Genome-wide maps of chromatin state have been made for numerous modifications in a variety of cell types. The resulting maps show that modifications often exist in specific combinations corresponding to unique functional genomic features. For example, trimethylation of histone H3 at lysine 4 (H3K4me3) and lysine 27 (H3K27me3) exists at the promoters of a subset of genes in ES cells [4], [5]. Such ‘bivalent’ genes tend to be associated with developmental functions and are repressed in ES cells, but poised for activation upon differentiation. A more recent study examined nine histone modifications in nine human cell types and found 15 chromatin states with distinct profiles of chromatin marks and functional enrichments [6]. Epigenetic modifications may also be antagonistic. In *Arabidopsis thaliana* the histone H2 variant H2A.Z and DNA methylation (DNAme) are mutually antagonistic [7]. DNA methylation is associated with repression while H2A.Z promotes transcriptional competence. Mutation of the PIE1 subunit of the Swr1 complex that deposits H2A.Z leads to genome-wide hypermethylation, while mutation of the MET1 DNA methyltransferase engenders opposite changes in DNA methylation and H2A.Z deposition. In addition to the examples described, coordinate regulation of epigenetic modifications has been demonstrated in a number of studies, consistent with the hypothesis of a histone code [8]–[11].

DNA methylation and H3K27me3 are both involved in the establishment and maintenance of epigenetic gene silencing. There are data showing coordinate regulation between the marks. Some evidence points toward a cooperative relationship. For example, the polycomb group protein EZH2 has been shown to positively regulate DNA methylation [12]. In these studies, EZH2 was observed to interact with DNA methyltransferases (DNMTs) and was required for DNA methylation of EZH2-target promoters. Alternatively, several lines of evidence suggest the coordination between DNAme and H3K27me3 may be antagonistic. A proteomic analysis has shown the PRC2 components EED and SUZ12 are excluded from methylated DNA [13], and in neural stem cells *Dnmt^3a^* deficiency leads to increased H3K27me3 [14]. Also, our lab has previously shown that at the imprinted locus *Rasgrf1* DNAme and H3K27me3 are mutually exclusive [15]. Finally, additional studies suggest that an important relationship between DNAme and H3K27me3 is disrupted in cancer cells. Polycomb group targets are more likely to have cancer-specific promoter DNA hypermethylation than non-targets [16]–[18]. However, embryonic carcinoma cells lack DNA hypermethylation at PRC targets [19], and knockdown of EZH2 in cancer cells may lead to hypomethylation [20]. Thus the evidence of interaction is conflicting, but it is clear that the relationship between these marks is important in both normal and cancerous cells.

Here, we attempt to address the relationship between DNAme and H3K27me3 by undertaking a genome-wide analysis to examine the effect loss of one mark has upon the placement of the other. We use mouse embryonic stem cells with defective PRC2 activity to examine the effect on the placement of DNAme, and use cells with defective DNA methyltransferase activity to identify regions with altered levels of H3K27me3. Finally, we perform RNAseq analysis in both cell types to try to associate gene expression changes with changes in the placement of either mark. We show that PRC2 activity is required for proper placement of DNAme at a number of developmentally important genes. We also demonstrate that DNAme is globally repressing the placement of H3K27me3. Our expression studies show that the coordinate regulation between these marks does not appear to have a direct effect on gene expression in the undifferentiated cells, but we show that the indirect effects on gene expression of loss of PRC2 or DNA methyltransferase have a remarkable similarity.

## Results

### Changes in DNAme in H3K27me3-deficient ES Cells

**Figure 1:**
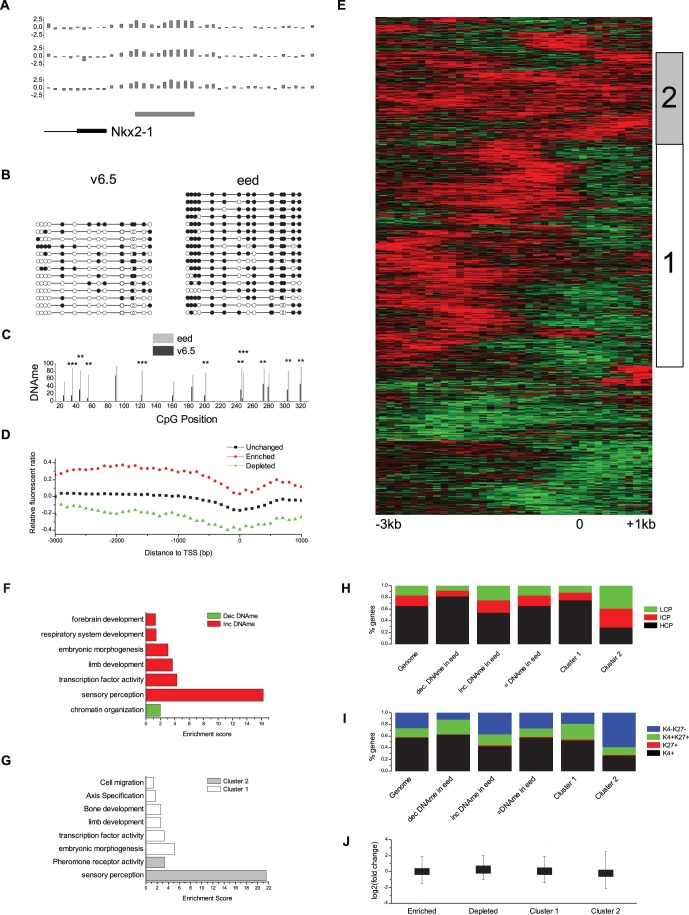
Loss of PRC2 activity leads to changes in DNA methylation. a, Relative fluorescence ratios for each probe from three independent MeDIP-chip experiments across the Nkx2-1 promoter. The peak of increased DNA methylation is indicated under the probes (grey bar) and the first 1 kb of the gene is indicated on the bottom. **b,** Validation of the peak of increased DNA methylation by bisulfite PCR. Each line represents an individual clone. Methylated CpGs are indicated by filled-in circles. **c,** A Fisher’s exact test was conducted for each CpG in (b) (** p<.01, *** p<.001). **d,** Profile of average DNA methylation relative to TSS calculated in 100 bp bins. **e,** Hierarchical clustering was performed on MeDIP-chip enrichment profiles to identify genes with similar profiles. 1,282 genes that passed the filtering step of the clustering software are on the y-axis. The x-axis is based on average fluorescent ratios in 100 bp bins from −3 kb (left) to +1 kb (right). Red indicates increased DNA methylation and green indicates decreased DNA methylation while black indicates unchanged DNA methylation. **f,** Gene ontology classifications for genes with increased (red) or decreased (green) DNAme. **g,** Gene ontology classifications for genes with increased DNAme upstream of the TSS (cluster 1, white) or across the entire promoter (cluster 2, grey). **h,** Classification of promoters based on CpG content. HCP, ICP & LCP, High-, Intermediate- & low CpG content promoter. **i,** Classification of promoters based on presence of H3K27me3 & H3K4me3. CpG and bivalent data used in (h) and (i) from [5] **j,** Boxplot of expression level change for genes enriched or depleted for DNAme in *Eed^−/−^* cells as well as for each of the two clusters described in (e).

In order to investigate changes in DNAme that occur as a consequence of loss of H3K27me3 we globally assayed DNAme using Methyl DNA immunoprecipitation followed by hybridization to a promoter microarray (MeDIP) in ES cells derived from *Eed^17Rn5–3354SB^* (*Eed^−/−^)* mutant mice. EED is one of the three components of the PRC2 complex and is required for normal H3K27 trimethylation. EED binds to histone tails carrying trimethyl-lysine residues and activates the methyltransferase activity of PRC2 [21]. Without EED, H3K27me3 is undetectable, while there is no difference in H3K9me3 [22]. We performed three independent MeDIP experiments and identified 2,296 regions with significant changes in DNAme as a consequence of loss of H3K27me3. Pairwise Pearson correlations showed good correlation of peak intensities between the three arrays (Figure S1). These peaks correspond to 2,933 promoters and 1,413 genes according to the Ensembl annotation of the NCBIM37 assembly of the mouse genome (Table S1). Of the 1,413 genes with changes in DNAme 861 showed increased DNAme and 552 showed decreased DNAme. Peaks were validated by sequencing >15 independent clones of PCR-amplified bisulfite-treated DNA and testing for changes in DNA methylation using a Fisher’s exact test (Figure 1A–C, Figure S2). In total, 7 peaks from 6 genes were validated. Interestingly 23 promoters showed peaks of both increased and decreased DNAme within the same promoter (Figure S2E-F), demonstrating that loss of PRC2 activity can have opposite effects on DNAme at close proximity, consistent with an earlier report of DNAme changes at the *Cdkn1c* and *Grb10* loci in *eed^−/−^* mice [23].

In order to examine whether changes in DNAme tend to happen in a focused location relative to the transcriptional start site (TSS) we aligned all genes with or without changes of DNAme and averaged the enrichment scores for all probes in 100-bp bins (Figure 1D). We see that changes in DNAme are distributed across the promoter with the greatest level of enrichment at between 1 and 2 kb upstream of the TSS. Gene ontology analysis of genes with changes in DNAme showed that genes with decreased DNAme in *Eed^−/−^* cells tended to be involved in chromatin organization while genes with increased DNAme were either involved in sensory perception or were developmentally important genes (Figure 1F). Genes with decreased DNAme also tended to be enriched for high-CpG-content (HCP) promoters and bivalent chromatin marks, while genes with increased DNAme tended to be genes with low- (LCP) or intermediate-CpG-content promoters (ICP) that lacked H3K4me3 and H3K27me3 in wildtype ES cells (Figure 1H,I). It is interesting to note that the lack of H3K27me3 in the promoter of genes with increased DNAme may indicate that H3K27me3 is regulating the placement of DNAme in an indirect manner. Hierarchical clustering of annotated mouse transcripts on the basis of DNAme patterns produced three main groups. One cluster had all of the genes with depleted DNAme, while the transcripts with increased DNAme were divided into two groups (clusters 1 & 2, Figure 1E). The first cluster had peaks of increased DNAme upstream of the TSS, while the second had increased DNAme across the entire promoter. These two clusters also corresponded to GO annotation and promoter CpG content in wildtype ES cells. Genes with increased DNAme across the promoter were genes with functions in sensory perception and pheromone receptor activity, had ICP and LCP promoters and lacked H3K4/K27 methylation, while genes with increased DNAme upstream of the promoter were developmental genes with HCP promoters and were enriched for bivalent chromatin marks (Figure 1G–I).

We performed RNAseq on wildtype and *Eed* ^−/−^ ES cells to determine if PRC2-dependent changes in DNAme led to expression level changes. While the gene ontology terms associated with genes with expression changes in *Eed^−/−^* cells are enriched for developmental functions, as previously shown (Table S2) [24], we saw no significant change in expression in genes which have H3K27me3-dependent changes in DNAme (Figure 1J), suggesting that coordinate regulation of DNAme levels by PRC2 is not directly controlling gene expression, at least in undifferentiated ES cells. However, we note that it is possible this coordination might poise genes for properly controlled expression after differentiation. Our work thus far demonstrates that the patterns of changes in DNAme that occur as a consequence of loss of PRC2 activity correlate with a particular epigenetic state in wildtype ES cells and with specific gene functions.

### DNAme Globally Antagonizes the Placement of H3K27me3

**Figure 2:**
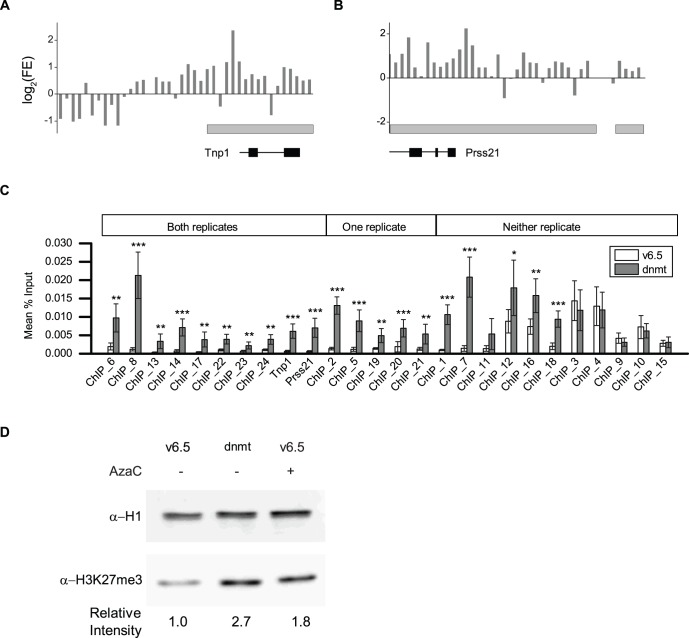
Increased H3K27me3 in *Dnmt^TKO^* ES cells. a, b, Average normalized fold enrichment of ChIP-seq reads in 100 bp bins in *Dnmt^TKO^* cells relative to wildtype for two representative loci, *Tnp1* (a) and *Prss21* (b). ChIP-seq peaks are indicated by the grey bars and genes are indicated at the bottom. **c,** qPCR validation of ChIP-seq peaks. Each bar represents the average percent input immunoprecipitated for six biological replicate ChIP experiments using chromatin from wild type v6.5 cells or *Dnmt^TKO^*. Note that all six ChIP experiments are independent of those used to generate the ChIP-seq libraries. Primers used in (c) are listed in Supplementary Table 4 (* p<.05, ** p<.01, *** p<.001). **d,** Western blot of acid-extracted histones from v6.5 (+ or - 5-AzaC) and *Dnmt^TKO^* cells. H1 is used as a loading control. Relative intensity of anti-H3K27me3 bands is shown on the bottom. Quantification was done using imageJ and normalized to H1.

As a reciprocal experiment we investigated the effect loss of DNAme had on the placement of H3K27me3 by performing ChIP-seq for H3K27me3 on cells with severely depleted DNA methyltransferase (DNMT) activity. DNMT triple-knockout cells (*Dnmt^TKO^*) lack genes for producing the two *de novo* DNMTs, DNMT3a and DNMT3b, and have the transcript of the maintenance DNMT, *Dnmt1,* depleted by stable expression of a shRNA [25]. The methylation level of these cells is 1.3% of that seen in wild type cells. We performed ChIP-seq on two biological replicates each for wildtype and *Dnmt^TKO^* cells (Figure 2A, B). A comparison with published datasets shows our ChIP-seq results are comparable to previously published H3K27me3 levels in both wildtype and *Dnmt^TKO^* cells (Figure S5). The first replicate generated 605,487 peaks of increased H3K27me3 in *Dnmt^TKO^* cells, covering 887,929,154 bp. The second replicate had 563,216 peaks covering 870,300,855 bp. On average, our ChIP-seq showed that H3K27me3 is increased on 32.4% of the mouse genome in the absence of DNA methylation. Intersection of the two replicates yielded 471,011 peaks covering 474,802,970 bp. We designed qPCR primers to ten peaks common to both replicates and tested six biological replicates each of wildtype and *Dnmt^TKO^* cells to validate the ChIP-seq results. All ten peaks confirmed the significantly increased H3K27me3 levels as assayed by qPCR (Figure 2C). Since we couldn’t identify a single false positive peak by qPCR, we validated an additional five peaks that were significant in one of our two replicates. All five of these peaks were also significantly enriched by qPCR (Figure 2C). If we examine the union of the two replicates, we get 756,847 peaks covering 1,218,250,739 bp, or ∼45% of the mouse genome with increased H3K27me3 in the absence of DNA methylation. Finally, we tested eleven sets of primers that covered regions that didn’t have a peak in either biological replicate. Interestingly, six of the eleven sets of primers showed significantly increased levels of H3K27me3 in *Dnmt^TKO^* cells. This suggests our ChIP-seq results may in fact be underestimating the portion of the genome with increased H3K27me3 in the absence of DNA methylation. We suspect peak calling programs have difficulty calling peaks when there are such high levels of enrichment across the genome. We confirmed the increase of H3K27me3 in *Dnmt^TKO^* by western blot and saw a nearly 3-fold increase in H3K27me3 in *Dnmt^TKO^* cells relative to wildtype cells (Figure 2D). This effect was also present in v6.5 cells treated with the DNA methyltransferase inhibitor 5-azacytidine. The pharmacological and genetic manipulations that impair DNAme both support the conclusion that DNAme has a potent negative influence on H3K27me3 in ES cells, regardless of their origin.

**Figure 3:**
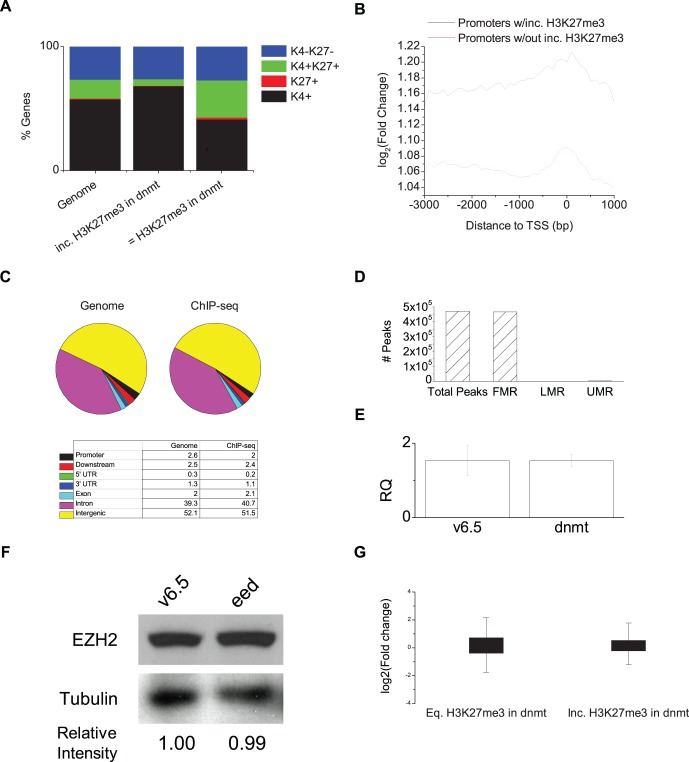
Global antagonism to H3K27me3 in *Dnmt^TKO^* cells. a, Classification of promoters identified in ChIP-seq experiment based on presence of H3K27me3 and H3K4me3 in wildtype cells. H3K4 and H3K27 methylation data from [5]. **b,** Profile of enrichment of ChIP-seq tags in 100 bp bins across the promoter for all genes with or without peaks of increased H3K27me3 in *Dnmt^TKO^* cells. **c,** Distribution of ChIP-seq reads according to genomic features. **d,** Number of ChIP-seq peaks intersecting with either fully-, low- or unmethylated regions according to data from [26]. **e,** Expression level of *Eed* in v6.5 and *Dnmt^TKO^* cells by qRT-PCR. **f,** Western blot analysis of EZH2 in v6.5 and *Dnmt^TKO^* cells. Relative intensity of EZH2 band from calculated using ImageJ is shown on the bottom. Intensity levels of EZH2 are normalized to Tubulin. **h,** Boxplot of expression level change for genes enriched in H3K27me3 in *Dnmt^TKO^* cells.

The 471,011 peaks present in both replicates show increased H3K27me3 at 50,659 annotated regions within transcripts from 20,254 gene promoters (−3 to +1 kb from the TSS), or about 55% of all known genes. The 756,847 peaks that represent the union of the two replicates intersect with 76,596 annotated transcripts from 30,037 gene promoters, or 82% of known genes. In contrast, only 861 gene showed increased DNA methylation in EED-deficient cells, demonstrating the antagonism of H3K27me3 placement by DNA methylation is far more widespread than the antagonism of DNA methylation by H3K27me3. Comparing the genes with increased H3K27me3 in *Dnmt^TKO^* cells with patterns of H3K27me3 in wildtype ES cells shows that the genes with increased levels of H3K27me3 are enriched for genes that lacked H3K27me3 in wildtype ES cells (Figure 3A). Enrichment of H3K27me3 appears to be evenly distributed across the promoter, with slightly increased levels of enrichment at the TSS (Figure 3B). Examining the distribution of peaks of increased H3K27me3 across the mouse genome shows a pattern indistinguishable from the genome in general (Figure 3C).

In order to examine if DNAme is antagonizing the placement of H3K27me3 by a direct mechanism we compared our data with published mouse wildtype ES cell methylome data. If DNAme is antagonizing H3K27me3 directly the sites of increased H3K27me3 in *Dnmt^TKO^* cells should contain DNAme in wildtype ES cells. We see that over 99% of the regions with increased H3K27me3 in *Dnmt^TKO^* overlap fully methylated regions in wildtype ES cells [26], consistent with the hypothesis that DNAme is globally antagonizing the placement of H3K27me3 (Figure 3D). It has been proposed that increased H3K27me3 in *Dnmt^TKO^* cells may be due to a compensatory effect [27]. Our RNAseq data showed no increase in *Eed* expression in *Dnmt^TKO^* cells (fold change = .91, p-value = 0.4). In order to confirm this we assayed for *Eed* expression in *Dnmt^TKO^* cells by qRT-PCR. We found no transcriptional upregulation of *Eed* in *Dnmt^TKO^* cells (Figure 3E). We also tested for increased PRC2 levels by western blot for EZH2 in *Dnmt^TKO^* cells. We found no change in the level of EZH2 protein in *Dnmt^TKO^* cells (Figure 3F). These results are consistent with the hypothesis that DNAme is directly antagonizing placement of H3K27me3 as opposed to some sort of compensatory effect.

To determine if loss of DNAme and accompanying acquisition of H3K27me3 affected gene expression in ES cells we again used RNAseq to see if genes with increased levels of H3K27me3 had concurrent changes in gene expression. As in the previous experiment, we do not see a change in expression in genes that have gained H3K27me3 as a consequence of disrupted DNA methyltransferase activity (Figure 2H), suggesting that coordinate regulation of H3K27me3 by DNAme is not directly controlling gene expression. Our ChIP-seq data demonstrate that DNA methylation is globally antagonizing the placement of H3K27me3 in wildtype ES cells by a direct mechanism.

### Similar Changes in the Transcriptional Program of *Dnmt^TKO^* and *Eed^−/−^* Cells

**Figure 4:**
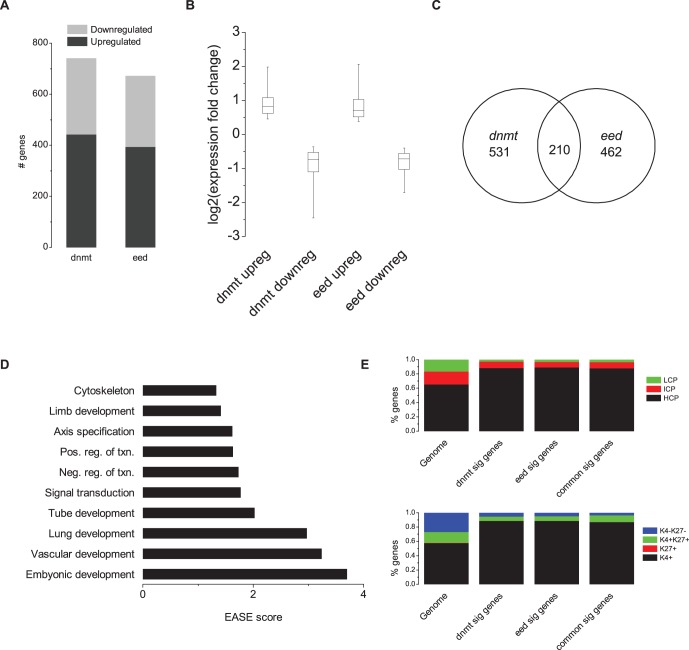
*Eed^−/−^* and *Dnmt^TKO^* cells have similar gene expression changes relative to wildtype cells by RNA-seq. **a,** Number of genes in *Dnmt^TKO^* and *Eed^−/−^* cells with significant changes in expression relative to wildtype cells. **b,** Boxplot of mean fold change in expression level relative to wildtype. **c,** Venn diagram showing number of genes with significant expression level changes common to both *Eed^−/−^* and *Dnmt^TKO^* cells. Significance of common genes determined by chi-square test, df = 1 (p<.0001). **d,** Gene ontology analysis of genes commonly misregulated in both *Eed^−/−^* and *Dnmt^TKO^* cells. **e,** Classification of genes commonly misregulated in *Dnmt^TKO^* and *Eed^−/−^* cells based on promoter CpG content, or H3K4me3 and H3K27me3 marks. Data from [5].

Although we could find no direct effect of coordinate regulation of DNAme and H3K27me3 on gene expression in ES cells, we used RNAseq to examine the effect loss of PRC2 or DNA methyltransferase activity has on gene expression generally. Our RNAseq results were validated by qRT-PCR. For eight of nine genes tested, qRT-PCR results agreed with genes identified as significantly differentially expressed by RNAseq (Figure S3). We found 741 genes with significant changes in *Dnmt^TKO^* cells relative to wildtype, similar to the 672 genes with a significant change in gene expression in *Eed^−/−^* cells (Figure 4A, Table S3). Also, a similar proportion of the changes are upregulation, 442 (60%) in *Dnmt^TKO^* and 394 (59%) in *Eed^−/−^*. The magnitude of the expression change is also similar between the two cell lines (Figure 4B). Upregulated genes average a fold change of 1.77 in *Dnmt^TKO^* and 1.8 in *Eed^−/−^* while downregulated genes average a fold change of 0.6 in *Dnmt^TKO^* and 0.61 in *Eed^−/−^*. Interestingly, a highly significant number of genes, 210, are changed in both cell types (Figure 4C). Of those the majority, 167, have gene expression changes that are consistent in both cell types. Interestingly, we see an equal number of genes being upregulated and downregulated. 83 genes are upregulated in both cell types while 84 genes are downregulated.

While the genes with significant expression changes in either cell type have a wide range of functions (Table S2), the gene ontologies of the genes being commonly regulated by *Dnmt^TKO^* and *Eed^−/−^* cells tend to be associated with developmental functions (Figure 4D). Regardless of the cell type, the genes with significant changes in gene expression tend to have HCP promoters containing H3K4me3 (Figure 4E). Our data are consistent with other studies that have shown that loss of DNAme does not cause massive deregulation [27], [28]. It is novel, however, to see that loss of PRC2 or DNA methyltransferase activity has similar consequences on gene expression, perhaps because both marks are required to control expression of a similar set of transcriptional regulators.

## Discussion

Our work shows that DNA methylation is globally antagonizing the placement of H3K27me3. It is well established that transposons and repeats are uniformly methylated, and generally believed that transposon defense is an ancient function of methylation [29]–[31]. In addition, in mammals and *A. thaliana* imprinted genes are regulated by DNAme [32], and the bodies of active genes are methylated [33]–[35]. Our data along with that of others [36] would suggest an additional role for DNA methylation in mammals may be to inhibit the inappropriate placement of histone modifications, specifically H3K27me3.

During the analysis of our data Brinkman et al. reported ChIP-seq for H3K27me3 in *Dnmt^TKO^* cells [27]. In their paper they report that total loss of DNAme is associated with alteration of H3K27me3 across the genome. They specifically reported broad local enrichments of H3K27me3 at megabase scale. Our data are consistent with their reported results. The authors hypothesize that the accumulation of H3K27me3 is due to a compensatory repressive effect instigated by the loss of DNA methylation. We propose that DNAme may be globally repressing the deposition of H3K27me3 by impairing an affinity that PRC2 has for unmethylated CpGs. Indeed, it has been shown that large GC-rich elements depleted of activating transcription factor motifs mediate PRC2 recruitment in mammals [37] and that methylation of DNA impairs binding of PRC2 in vitro [14]. It is possible that the presence of DNA methylation across most of the mammalian genome is inhibiting an inherent affinity that PRC2 has for CpG-rich sequences. Further experiments will be needed to clarify how DNAme is acting to inhibit H3K27me3.

In addition to global antagonism of H3K27me3 by DNAme, we also show that H3K27me3 is required for proper placement of DNA methylation. When PRC2 activity is lost we see both increases and decreases in DNA methylation within the promoters of primarily developmentally important genes. Our original intent in examining DNAme and H3K27me3 was to determine if mutual antagonism between the two marks that we have previously shown at the *Rasgrf1* imprinted locus is a general rule operating genome wide. We identified 439 genes that had increases of DNAme upon loss of PRC2 activity, as well as increases of H3K27me3 upon loss of DNA methyltransferase activity. This set of genes does not appear to be enriched for genes with expression changes in either *Dnmt^TKO^* or *Eed^−/−^* cells (data not shown), suggesting that coordinate regulation between DNAme and H3K27me3 is not directly controlling gene expression within undifferentiated ES cells.

Since many genes undergoing DNAme changes upon loss of H3K27me3 have high CpG-content promoters, contain bivalent epigenetic marks, and are linked to developmental GO terms, we hypothesized that exclusion of DNAme by H3K27me3 might be required to create chromatin states permissive for subsequent expression upon differentiation. This would explain why these genes show no changes in expression in the mutant ES cell lines. Unfortunately, although *Eed^−/−^* is dispensable for the maintenance of pluripotent stem cells, it is required for the differentiation and/or maintenance of multipotent progenitors, limiting our ability to differentiate *Eed^−/−^* cells in vitro [24], [38]. To explore this possibility, we compared the list of genes with altered DNAme caused by loss of H3K27me3, to published datasets describing gene expression differences in neural progenitor cells (NPCs) or MEFs relative to ES cells [39]. Although genes undergoing DNAme changes upon loss of H3K27me3 were not activated in either NPCs or MEFs (data not shown), this does not preclude the possibility that coordinated regulation of PRC2 and DNAme controls gene expression in other differentiated cell types.

Recently, it has been demonstrated that within the gene bodies of HCC1954 cells there is mutual antagonism between H3K27me3 and DNAme in an allele specific manner, and that disruption of this mutual antagonism leads to misexpression [40]. We are not able to complete a similar analysis because our MeDIP experiments do not provide information about allele-specific methylation or data across the gene body. It is possible that by comparing our ChIP-seq dataset with a more complete analysis of DNAme in *Eed^−/−^* cells derived from polymorphic strains that we may be able to identify important examples of mutual antagonism between DNAme and H3K27me3.

Finally, We looked at the expression changes that occur upon loss of either PRC2 or DNA methyltransferase activity and find an interesting overlap in regulation at a number of developmentally important genes and similar effects on gene expression upon loss of either mark. We do not find, however, that the genes that are regulated by crosstalk between these two epigenetic marks correlate with genes that have expression changes in either cell type, so it appears that the crosstalk between the marks is not directly controlling gene expression, at least in undifferentiated ES cells. Future studies examining the effect loss of one epigenetic mark has on the placement of other marks will be important to understand how coordinate regulation of epigenetic modifications effects gene regulation. It is possible that using a similar approach to examine additional marks coordinately regulated with H3K27me3, such as H3K4me3, or with DNAme, such as H3K9me3, will give important insights into gene regulation. Additionally, as high-throughput sequencing technologies continue to increase read number and read length, comprehensive methylome analysis will become more common, giving a greater ability to intersect ChIP-seq datasets with methylome data genome-wide with basepair resolution.

## Materials and Methods

### Cell Culture

V6.5 [41], *Eed^−/−^* [38] & *Dnmt^TKO^* [25] murine ES cells were cultivated in 5% CO_2_ at 37°C on irradiated MEFs in DMEM containing 15% FCS, LIF, Penicillin/streptomycin, L-glutamine, NEAA, L-glutamine & 2-mercaptoethanol. Cells were passed at least 2 times feeder-free on 0.2% gelatin to exclude feeder contamination. The DNMT deficiency in *Dnmt^TKO^* cells was validated by qRT-PCR. The inactivating mutation in the *Eed* ^−/−^ cell line was validated by sequencing, and low EED protein levels confirmed by western blot (Figure S4). For 5-AzaC treatment, v6.5 cells were grown to 60% confluency then treated with 30 ng/mL 5-AzaC for 72 hours prior to preparing histones.

### Methyl-DNA Immunoprecipitation

MeDIP was performed as described [42], [43]. Briefly, DNA prepared from v6.5 and *Eed* ^−/−^ cells at passage 13 and 6, respectively, was treated with RNase A, incubated at 37°C for 30 minutes, sonicated on a Covaris sonicator and isopropanol precipitated. 4 ug Sonicated DNA was incubated at 4°C overnight with 10 ug anti-5-MeC antibody (Eurogentec, BI-MECY-0100) in IP buffer (10 mM Na-Phosphate pH7, 140 mM NaCl, 0.05% Triton). Samples were then incubated with anti-mouse M-280 Dynabeads (Invitrogen, 112.01D) for 2 hours in 0.1% BSA/PBS at 4°C. Samples were washed twice, treated with Proteinase K, extracted with 1∶1 phenol/chloroform & ethanol precipitated. 100 ng of each sample was then amplified using the GenomePlex Complete Whole Genome Amplification kit (Sigma, WGA2) and hybridized to a NimbleGen 3×720 K CpG Island Plus RefSeq Promoter Array (NimbleGen, 05924537001). 20,404 promoters are on the array. The Cornell Life Sciences Core Laboratory performed array hybridization. Peaks were called using the NimbleGen Nimblescan software with the default settings. We defined a gene promoter as the region from −2 kb to +1 kb of a transcriptional start site according to the Ensembl annotation of the mm9 version of the mouse genome.

### Bisulfite Sequencing of PCR Fragments

Bisulfite conversion was performed using the MethylEasy Xceed kit (Human Genetic Signatures, ME002). PCR products were gel purified and cloned using the TOPO TA Cloning Kit (Invitrogen, 45–0641). Primers used are listed in Supplementary Table 4. At least 15 independent clones were sequenced and analyzed using the QUMA platform (http://quma.cdb.riken.jp/).

### Cluster Analysis and Binning of Microarray Data

Clustering of microarray data was done using Cluster 3.0 [44]. Microarray data were reformatted for the Cluster program using custom perl scripts. Data from all three arrays were intersected using BEDTools [45] and binned using R. Data were visualized using Origin.

### ChIP-seq

2×15 cm dishes of ES cells were fixed in 1% formaldehyde/PBS for 30 minutes at room temperature. Fixing was quenched with 1.4 mL 2.5 M Glycine. Cells were scraped into a 15 mL tube, washed twice with PBS and stored at −80°C. Chromatin was prepared by incubating cells 10 minutes at 4°C in lysis buffer 1 (50 mM Hepes, pH 7.5, 140 mM NaCl, 1 mM EDTA, 10% Glycerol, 0.5% NP-40, 0.25% Triton w/protease inhibitors) followed by a 20-minute incubation at room temperature in lysis buffer 2 (200 mM NaCl, 1 mM EDTA, 0.5 mM EGTA, 10 mM Tris pH 8 w/protease inhibitors). Cells were resuspended in 500 uL buffer 3 (1 mM EDTA, 0.5 mM EGTA, 10 mM Tris pH 8 w/protease inhibitors) and sonicated on a Misonix 3000 sonicator for 90 seconds (30 seconds on/30 seconds off). 350 uL of sonicated chromatin was used to create ChIP-seq libraries as previously described [46].

### ChIP-seq Analysis

Chip-seq data were analyzed for quality control using the FastX Toolkit [47](http://cancan.cshl.edu/labmembers/gordon/fastx_toolkit/). Mapping was done using Bowtie and peaks called using MACS with *Dnmt^TKO^* as the treatment group and v6.5 as the control group, using the mm9 version of the mouse genome as the reference [48], [49]. Meta-analysis was done using CEAS [50].

### qPCR

Unamplified DNA from six independent ChIP experiments was used for qPCR on an ABI7500. Primers used are in Table S4. Percent input was calculated using absolute quantification based on a standard curve made from input DNA. Validation of RNAseq results by qRT-PCR was done using primers to *Actin* and *Rpl32* as endogenous controls.

### RNAseq

mRNA-seq libraries were created as previously described [51]. RNA-seq reads were processed with the FastX Toolkit and mapped with TopHat [52]. RNA-seq reads were 83 bp after processing. The Cufflinks program was used to calculate RPKM expression values [52]. Significantly differentially expressed genes were identified as those called by both Cuffdiff [53] and DESeq [54] using the default settings.

### Western Blot

2 ug acid-extracted histones or 10 ug cell extract were run on a 15% (histones) or 10% (cell extract) SDS-PAGE gel and transferred to nitrocellulose. Membranes were blocked with TBST +5% milk and incubated with primary antibody and secondary antibodies for two hours at room temperature in TBST +1% milk. Primary antibody concentrations were 1∶1000 for H3K27me3 (Active Motif 39155), H3K9me3 (Active Motif 39162), H1 (Active Motif 39707), EZH2 (Cell Signaling 3147) & Tubulin (Abcam 16504). Secondary antibody concentrations were 1∶10,000 (Millipore 12–348 & Abcam 102448). Chemiluminescent detection of HRP was done using the SuperSignal West Dura Extended Duration Substrate (Thermo 34075).

## Supporting Information

Figure S1 **Correlation of log2(fold enrichment) values from MeDIP arrays. a, b, c,** Scatterplots of fluorescent intensity ratios from each array. 10,000 probes were randomly chosen to plot out of 720,000 on the array. Each probe is represented with a single dot set at 90% transparency. **d,** R-values from Pearson correlation test of fluorescence intensity ratios for all probes on each slide.
(EPS)

Figure S2 **Validation of MeDIP array data by bisulfite PCR.** Validation of peaks of changed DNA methylation in *Eed^−/−^* cells by bisulfite PCR. Each line represents an individual clone. Methylated CpGs are indicated by filled-in circles. Beneath each set of methylation data is a fisher’s exact test that was conducted for each CpG (* p<.05, ** p<.01, *** p<.001). Genes validated are **a**, *Tdrd5*, **b**, *Pdgfrl*, **c**, *Gas5*, **d**, *Fbxo18*, **e**, **f**, *Fv1*. Note that *Fv1* is a gene with peaks of both increased (e) and decreased (f) DNAme in the promoter.
(EPS)

Figure S3 **Validation of genes called as differentially expressed in** ***Dnmt^TKO^*** **and** ***Eed^−/−^*** **cells by RNAseq. a,** RPKM values calculated by Cufflinks for genes used in qRT-PCR in (b). A box has been placed around all the genes called as significantly differentially expressed by both Cuffdiff and DEseq. **b,** Average relative quantification (RQ) by qRT-PCR for genes called as significantly differentially expressed. Error bars are based on 4 independent biological replicates (* p<.05, ** p<.01, *** p<.001). *Ptprk*, *Rab7* and *Cpne3* are negative controls.
(EPS)

Figure S4 **Validation of** ***Dnmt^TKO^*** **and** ***Eed^−/−^*** **cell lines. a,** qRT-PCR for transcripts of the three mammalian DNA methyltransferases, *Dnmt1, Dnmt3a* & *Dnmt3b*, in v6.5, *Eed^−/−^*, and *Dnmt^TKO^* cells. **b,** Sequencing results from v6.5 and *Eed^−/−^* cells lines. *Eed^l7Rn5–3354SB^* contains a T to C transition at position 1038 leading to a Leu (CTG) to Pro (CCG) change [55]. **c**, Western blot for H3K27me3 in v6.5 and *Eed^−/−^* cell lines. H3K9me3 is used as a loading control.
(EPS)

Figure S5 **Comparison of ChIP-seq results to published datasets.** ChIP-seq results in blue. Published data is in red from [27] **a,** Wig profile showing number of reads across the Pparg locus spanning 150 kb. A comparable profile of reads across Pparg is also shown in [5]. **b,** Wig profile across 300 kb span of chromosome 11 spanning 12 genes. This region is also shown in [5]. **c**, Wig profile across 2 mb span of chromosome 4. Large region of increased H3K27me3 in *Dnmt^TKO^* cells is also shown.
(EPS)

Table S1 List of MeDIP-chip peaks with changes in DNAme in *Eed^−/−^* cells relative to v6.5 cells. Transcript id’s, gene id’s, gene names and coordinates are according to the Ensembl annotation of the NCBIM37 version of the mouse genome.
(XLS)

Table S2 Gene ontology terms associated with genes with expression changes in *Eed^−/−^* or *Dnmt^TKO^* cells.
(XLS)

Table S3 1,413 genes with changes in gene expression in *Eed^−/−^* or *Dnmt^TKO^* according to RNAseq analysis. Results include the output from both Cuffdiff and DESeq.
(XLS)

Table S4 PCR primers used in this study.
(XLS)
